# Anxiety, depression, and concentration in cancer survivors: National Health and Nutrition Examination Survey results

**DOI:** 10.1007/s00520-023-07710-w

**Published:** 2023-04-15

**Authors:** Joanna E. Fardell, Chase M. Irwin, Janette L. Vardy, Melanie L. Bell

**Affiliations:** 1 Sydney Medical School, Faculty of Medicine and Health, University of Sydney, Sydney, Australia; 2 UNSW Medicine & Health, School of Clinical Medicine, UNSW Sydney, Kensington, Australia; 3 Western Sydney Youth Cancer Service, Westmead Hospital, Sydney, Australia; 4 Kids Cancer Centre, Sydney Children’s Hospital, Level 1 South, Randwick, Sydney, Australia; 5 Department of Epidemiology and Biostatistics, Mel and Enid Zuckerman College of Public Health, University of Arizona, Tucson, AZ 85724 USA; 6 Concord Cancer Centre, Concord Repatriation General Hospital, Sydney, New South Wales Australia

**Keywords:** Anxiety, Cancer, Cancer survivors, Concentration, Depression, Oncology

## Abstract

**Purpose:**

We report on prevalence of anxiety, depression, and concentration difficulties and their associations in survivors of cancer in a nationally representative sample up to 25 years after diagnosis.

**Methods:**

Using the National Health and Nutrition Examination Survey (NHANES) data from 2015 to 2018, participants between the ages of 18 and 79 self-reported on cancer history, symptoms of anxiety, depression, and difficulties with concentration.

**Results:**

Of 10,337 participants, 691 (6.7%) reported a previous diagnosis of cancer; the median time since diagnosis was 8 years. Prevalence was similar between those with and without cancer for anxiety (45.8% versus 46.9%) and depression (19.7% versus 20.0%). Concentration difficulties were more common (11.3% versus 9.0%) for those with a history of cancer compared to those without (adjusted OR = 1.38, 95% CI: 1.00–1.90). Prevalence of mental health symptoms was not related to time since diagnosis. Anxiety and depression were highly correlated (*r* = 0.81, 95% CI: 0.74–0.86) and moderately correlated with difficulty with concentration (*r* = 0.52, 95%CI: 0.40–0.64 and *r* = 0.64, 95% CI: 0.53–0.74 respectively).

**Conclusions:**

Difficulty with concentration was more commonly reported by participants with than without a cancer history. Report of anxiety and depression was no different between participants with and without a history of cancer. Anxiety, depression, and difficulties with concentration were strongly related. Further research is needed to explore if there is a causal association, and if so, the direction of these correlations, so that interventions may be appropriately targeted.

**Supplementary Information:**

The online version contains supplementary material available at 10.1007/s00520-023-07710-w.

Cancer survivors are at risk of physical and psychological morbidity in the years after treatment completion. Survivors experience anxiety, depression, and reduced cognitive function at rates greater than the general population. For example, 14–24% experience symptoms consistent with clinical levels of depression [1–3], and approximately 10% of survivors report clinical levels of anxiety [2]. In comparison, population estimates of depression and anxiety in the last year are 5% and 7% respectively [3–5]. Up to 75% of survivors also report difficulties with cognitive function, a rate higher than that detected by objective neuropsychological assessment [6–8]. Experiencing anxiety, depression, and decreased cognitive function impacts quality of life after cancer. Survivors reporting impaired memory and concentration experience difficulties returning to previous roles and work [8], greater reliance on psychotropic medication [9], and increased sleep disturbance [8]. Similar functional impacts are reported by survivors experiencing anxiety and/or depression [5, 10].

Some risk factors for increased anxiety, depression, and cognitive impairment appear to be shared. Survivors who are younger at diagnosis and female, and have lower education are more likely to report anxiety, depression, and impaired cognitive function [8, 9, 11, 12]. Older age at diagnosis is protective against anxiety and depression in the long term [12], but may be a risk factor for increased self-report of cognitive difficulties [13]. Those who are further from treatment appear to experience less depression [14], and self-reported cognitive difficulties [15], though anxiety may be more persistent over time [14]. However, there may be important differences in this trajectory according to cancer type, stage, and treatments received.

Self-reported cognitive symptoms, anxiety, and depression are strongly related [7, 16, 17]. The shared profile of risk factors has led some researchers and clinicians to consider the co-occurrence of mental health difficulties (i.e., anxiety, depression, and cognitive impairment) as a psychoneurological symptom cluster after cancer [16, 18]. To date, most research has focused on survivors of breast cancer. Research at the population level documenting the occurrence, and co-occurrence of mental health concerns across survivors of all cancers is scarce [15]. The aim of this study was to investigate whether cancer survivors have greater self-reported mental health symptoms (anxiety, depression, concentration difficulties) than individuals without a history of cancer using a population-based sample from the USA. A secondary aim was to investigate the effect of time since diagnosis for mental health symptoms among cancer survivors.

## Methods

### Study design and setting

National Health and Nutrition Examination Survey (NHANES) is a program of studies designed to assess the health and nutritional status of adults and children in the USA. The program has been administered every 2 years since 1999 and combines interviews and physical examinations. Data are collected on a voluntary basis and all results are either self-reported responses to interview questions or quantitative measurements administered by trained medical personnel. The survey examines a nationally representative sample of about 5000 persons through complex survey sampling. Oversampled subgroups include Hispanic persons, non-Hispanic Black persons, non-Hispanic Asian persons, and persons at or below the 185% of the US federal poverty line. Participants from the 2015–2016 and 2017–2018 datasets who were between the ages of 18 and 79 and recorded responses to mental health outcomes, age, gender, race/ethnicity, education, and cancer history were included in the analysis. All participants gave informed consent and ethical approval was granted by the National Center for Health Statistics Research Ethics Review Board Protocol #2011–17.

### Measures

The primary outcomes for this analysis are three mental health outcomes: anxiety (How often do you feel worried or anxious?); depression (How often do you feel depressed?); and concentration difficulties (Do you have serious difficulty concentrating?). These items are taken from the Disability Questionnaire, as developed by the National Center for Health Statistics of the Centers for Disease Control and Prevention. Development involved input from federal agencies, and academic and private researchers. Questionnaires were tested in the NCHS Questionnaire Design Research Laboratory and in field tests. For more information, see https://www.cdc.gov/nchs/nhis/nhis_disability.htm. The anxiety and depression outcomes were dichotomized as either *at least once a month* or *less than once a month* and the concentration difficulties outcome remained as either *yes* or *no*. The exposure of interest was past history of cancer or malignancy (Have you ever been told by a doctor or other health professional that you had cancer or a malignancy of any kind?) and was dichotomized as either *yes* or *no*. Non-melanoma skin cancer was excluded from the history of cancer group.

### Statistical methods

All analyses accounted for the complex survey design (clustering, stratification, weighting) using survey procedures available in SAS version 9.4 (SAS Institute, Cary, NC), using NHANES guidelines [19]. Descriptive statistics were stratified by cancer history. All percentages reported were survey-weighted. For the primary analysis, each mental health outcome was assessed through one unadjusted and two adjusted logistic regression models resulting in a total of nine models. Adjusted models included covariates selected a priori based on background knowledge and literature review. Covariates included age, gender (male/female), race/ethnicity (Hispanic, non-Hispanic White, non-Hispanic Black, and Other), and education (at least some college, no college). The first adjusted model estimated the effects of any type of cancer; the second modeled each tumor type. We categorized age into four separate strata: 18–35, 36–50, 51–65, 66–79 years.

Odds ratios (OR) and 95% confidence intervals (CI) were reported with the unexposed group being used as the reference group. Likelihood ratio tests were used to determine whether gender modified the relationship between cancer exposure and mental health outcomes. If the likelihood ratio test produced a statistically significant interaction, we stratified by gender and refit the models. Logistic regression was also used for our secondary analysis investigating the effect of self-reported time since cancer diagnosis among persons with a history of cancer. Polychoric correlation between each pair of mental health outcomes among persons with a history of cancer was also undertaken.

We performed a sensitivity analysis using propensity score stratification to account for large covariate imbalances (standardized mean differences > 0.25) in our study sample [20]. The propensity score model included gender, age, and race/ethnicity and accounted for the complex survey design using the methods outlined in Dugoff et al. [21]. Results from the sensitivity analysis were compared with the primary analysis to assess the robustness of the adjusted logistic regression models.

## Results

Of the 10,337 participants used for our analysis, 691 (6.7%) reported a previous history of cancer, excluding those with non-melanoma skin cancer (Table 1). As compared to participants with no history of cancer, participants with a history of cancer were more likely to be female (58% versus 51%), older (median = 63 versus 45 years), non-Hispanic White (76.5% versus 61.3%), and of slightly higher education (69% versus 64% with some college or more). Participants with a history of cancer were more heavily concentrated in the 51–65 (34.1%) and 66–79 (43.7%) age groups. The median time since diagnosis was 8 years (interquartile range, IQR = 4, 16) and median age at diagnosis was 51 years (IQR = 36, 61).

**Table 1:** Descriptive statistics of the NHANES Cancer Study, 2015–2018 (*N* = 10,337). Count data and survey-weighted percentages are presented

	Cancer history (*N* = 691)	No cancer history (*N* = 9646)
Gender, *N* (%)^a^		
Male	308 (42.0)	4674 (49.0)
Female	383 (58.0)	4972 (51.0)
Race/ethnicity, *N* (%)^a^		
Hispanic^b^	148 (8.3)	2732 (16.5)
Non-Hispanic White	318 (76.5)	2911 (61.3)
Non-Hispanic Black	136 (7.2)	2204 (11.8)
Other^c^	89 (7.9)	1799 (10.3)
Education, *N* (%)^a^		
No college	270 (30.6)	4232 (36.3)
Some college	421 (69.4)	5414 (63.6)
Age, *N* (%)^a^		
18–35	30 (5.7)	2881 (32.8)
36–50	91 (16.5)	2511 (27.1)
51–65	244 (34.1)	2751 (27.4)
66–79	326 (43.7)	1503 (12.6)
Age (years), median (IQR)	62.5 (51.9, 70.2)	44.5 (31.0, 57.8)
Age at diagnosis, median (IQR)	50.6 (36.4, 60.7)	
Years since diagnosis, median (IQR)	8.4 (3.6, 16.3)	
Tumor type, *N* (%)^a^		
Genitourinary	174 (20.8)	
Colorectal	54 (5.5)	
Upper gastrointestinal	22 (2.2)	
Hematological	36 (4.3)	
Respiratory	21 (1.7)	
Breast	131 (21.9)	
Gynecological	107 (16.6)	
Melanoma	58 (13.9)	
Other^d^	88 (13.0)	
Non-melanoma skin	NA	152 (100.0)

*Abbreviation*: *IQR*, interquartile range

^a^Percentages reported are survey-weighted

^b^Self-identified as Mexican or Other Hispanic

^c^Self-identified as Non-Hispanic Asian or Other

^d^Self-reported brain, nervous system, larynx/windpipe, mouth/tongue/lip, bone, soft tissue, or thyroid cancer

**Fig. 1:**
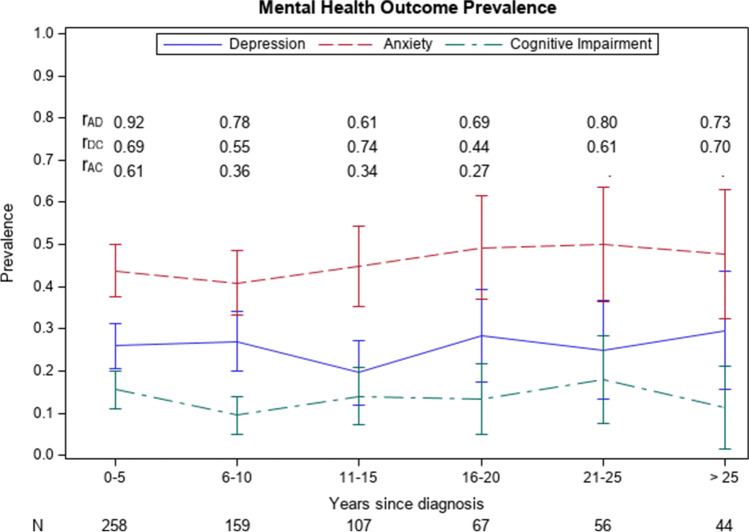
Proportion of participants with a history of cancer (*N* = 691) reporting anxiety, depression, and difficulty concentrating across time since diagnosis. Error bars are 95% CI. rAD shows the correlation between anxiety and depression across time since diagnosis, rDC shows the correlation between depression and difficulty concentrating, and rAC shows the correlation between anxiety and difficulty concentrating. Missing correlations in rAC are due to zero cells in the 2 × 2 table

Tables  2 shows the results from the primary analysis as well as the proportion of participants with negative mental health outcomes within each exposure group. Anxiety was reported in 45.8% of participants with a history of cancer, and 46.9% of participants with no history of cancer. Prevalence of depression was similar between the groups (19.7% and 20%) and concentration difficulties were slightly higher in those with a history of cancer, 11.3% versus 9.0%. Prevalence of each of the outcomes prior to dichotomization is given in supplementary table S2. A high correlation between outcomes was found among cancer survivors. The overall correlation between anxiety and depression was 0.81 (95% CI: 0.74–0.86); between anxiety and concentration difficulties 0.52 (95% CI: 0.40–0.64); and between depression and concentration difficulties 0.64 (95% CI: 0.53–0.74). Figure 1 shows the correlation between anxiety, depression, and reported concentration difficulties across each 5-year interval since diagnosis.

**Table 2:** Association between cancer history and experiencing mental health outcomes at least once a month in participants of NHANES, 2015–2018 (*N* = 10,337). Results of logistic regression analyses are presented with unadjusted and adjusted^b^ odds ratios (OR)

History of cancer/tumor type	Negative mental health outcomes^a^ *N* (%)	Unadjusted OR (95% CI)	Adjusted OR (95% CI)	Adjusted^b^ OR (95% CI)
Anxiety				
No cancer history	3964 (46.9)	Ref	Ref	Ref
Cancer history	308 (45.8)	0.95 (0.77, 1.17)	1.12 (0.87, 1.43)	
Genitourinary	61 (35.5)			1.12 (0.62, 2.00)
Colorectal	23 (52.0)			1.21 (0.67, 2.20)
Upper gastrointestinal	11 (70.3)			2.18 (1.13, 4.21)*
Hematological	14 (32.2)			0.59 (0.23, 1.55)
Respiratory	10 (51.8)			1.66 (0.81, 3.42)
Breast	58 (53.1)			1.27 (0.80, 2.04)
Gynecological	69 (59.4)			1.45 (0.81, 3.42)
Melanoma	21 (29.4)			0.55 (0.30, 1.03)
Other	41 (46.8)			1.10 (0.51, 2.39)
Depression^d^				
No cancer history	1909 (19.7)	Ref	Ref	Ref
Cancer history	177 (20.0)	1.02 (0.81, 1.28)	1.11 (0.87, 1.41)	
Genitourinary	37 (20.4)			1.48 (0.85, 2.59)
Colorectal	15 (20.5)			1.14 (0.44, 2.97)
Upper gastrointestinal	10 (45.0)			3.66 (1.28, 10.5)*
Hematological	8 (13.8)			0.71 (0.21, 2.34)
Respiratory	8 (32.0)			1.92 (0.47, 7.75)
Breast	28 (18.3)			0.88 (0.45, 1.72)
Gynecological	40 (26.7)			1.25 (0.64, 2.46)
Melanoma	11 (12.7)			0.73 (0.31, 1.73)
Other	20 (17.6)			0.93 (0.46, 1.89)
Concentration difficulties				
No cancer history	934 (9.0)	Ref	Ref	Ref
Cancer history	94 (11.3)	1.30 (0.96, 1.77)	1.38 (1.00, 1.90)*	
Genitourinary	14 (7.35)			1.00 (0.39, 2.55)
Colorectal	11 (18.9)			2.40 (1.07, 5.37)*
Upper gastrointestinal	3 (26.0)			3.94 (0.67, 23.1)
Hematological	8 (12.0)			1.49 (0.45, 4.97)
Respiratory	2 (3.2)			0.24 (0.04, 1.35)
Breast	21 (16.3)			2.07 (1.02, 4.20)*
Gynecological	19 (14.6)			1.43 (0.68, 3.00)
Melanoma	2 (1.5)			0.21 (0.04, 1.07)
Other	14 (10.8)			1.29 (0.50, 3.34)

^*^p < 0.05

^a^Percentages reported are survey-weighted

^b^Adjusted for gender identity, age, race/ethnicity, and education

^c^Self-reported anxiety occurring daily, weekly, or monthly

^d^Self-reported depression occurring daily, weekly, or monthly

^e^Self-reported difficulty concentrating

Statistically significant results were not found in any unadjusted models, although 95% CIs included potentially important values for each outcome. As compared to participants without a history of cancer, participants with a history of cancer had a higher adjusted odds of concentration difficulties (adjusted odds ratio (AOR) = 1.38, 95% CI: 1.00, 1.90). Adjusted odds were slightly elevated, but not statistically significant for depression (AOR 1.10 (95% CI: 0.86, 1.41)) and anxiety (AOR 1.12 (95% CI: 0.87, 1.43)) for persons with a history of cancer compared to those without. When comparing specific tumor types with no history of cancer, there was no evidence of an effect, except for upper gastrointestinal tumors for anxiety (AOR = 2.18, 95% CI 1.13, 4.21) and depression (AOR = 3.66, 95% CI 1.28, 10.5); and colorectal cancer and breast cancer for concentration difficulties (colorectal cancer AOR = 2.40, 95%CI 1.07, 5.37; and breast cancer AOR = 2.07, 95%CI 1.02, 4.20). No specific tumor types showed significant results for more than one mental health outcome. Given the small numbers in many of the cells by tumor type, care should be taken when interpreting the results. Gender modified the effect of cancer history on concentration difficulties (*p*_interaction, adjusted_ = 0.04), with larger effects seen for women (AOR = 1.66, 95% CI: 1.16, 2.37) than men (AOR = 0.9495% CI: 0.56, 1.58). There was no evidence of effect modification by gender for anxiety (*p*_interaction, adjusted_ = 0.23) or depression (*p*_interaction, adjusted_ = 0.64).

Table 3 shows the results from the secondary analysis which investigated the time since diagnosis among cancer survivors. Figure 1 shows similar proportions of survivors report anxiety, depression, and difficulty with concentration across each 5-year interval since diagnosis. No statistically significant results were discovered in unadjusted or adjusted models for anxiety (AOR for each 10 years since diagnosis = 0.98, 95% CI: 0.74, 1.30), depression (AOR = 0.90, 95% CI: 0.61, 1.35), or concentration difficulties (AOR = 0.77, 95% CI: 0.49, 1.20). Comparisons by tumor type in adjusted models did not yield any statistically significant results.

**Table 3:** Association between time since cancer diagnosis^a^ and mental health outcomes in participants with a history of cancer in NHANES, 2015–2018 (*N* = 691). Results of logistic regression analyses are presented with unadjusted and adjusted^b^ odds ratios (OR)

Outcome	Unadjusted OR (95% CI) for 5 years	Adjusted^b^ OR (95% CI) for 5 years
Anxiety^c^	0.97 (0.87, 1.09)	0.98 (0.86, 1.11)
Depression^d^	0.94 (0.80, 1.10)	0.95 (0.79, 1.14)
Concentration difficulties^e^	0.88 (0.70, 1.10)	0.86 (0.67, 1.10)

NB reference group is no cancer history

^a^Unit of time since cancer diagnosis evaluated for every 5 years

^b^Adjusted for gender identity, age, race/ethnicity, education, and tumor type

^c^Self-reported anxiety occurring daily, weekly, or monthly

^d^Self-reported depression occurring daily, weekly, or monthly

^e^Self-reported difficulty concentrating

Results from the sensitivity analysis mirrored those of the primary analysis. Supplementary Table 1 presents the results from the adjusted models in our sensitivity analysis and compares the results to the adjusted models of our primary analysis.

## Discussion

This paper reports on data from 10,337 participants in NHANES, 2015–2018, comparing those with a history of cancer to those without cancer on self-reported mental health outcomes. We found comparable rates of self-reported anxiety (around 46%) and depression (around 20%) between participants with and without a history of cancer. Overall, 11% of cancer survivors reported difficulties with their concentration compared to 9% of participants without a history of cancer. Cancer survivors had a 38% increased odds of self-reporting problems with their concentration compared with participants without a history of cancer. Colorectal and breast cancer survivors had a twofold increased odds of reporting difficulties concentrating compared to participants without a history of cancer. Women with a history of cancer were at higher odds of reporting cognitive symptoms compared to men with a history of cancer. Time since diagnosis did not impact the odds of reporting mental health concerns. There were strong correlations between mental health outcomes, particularly between anxiety and depression.

Anxiety and depression were commonly reported in our sample, regardless of cancer history, in contrast to previous studies identifying a difference in prevalence of symptoms between cancer survivors and controls or normative data [1–3, 12, 14]. We found approximately 20% and 46% of participants reported some degree of depression or anxiety at least once a month. This may be due to the sample and differences in measures employed. NHANES is a nationally representative sample for the USA, and most previous studies have not been population-based. Further systematic reviews including studies with validated and clinical measures indicate approximately 20% and 10% of cancer survivors have symptoms consistent with a diagnosis of depression or anxiety respectively [2, 14]. Participants with a history of upper gastrointestinal cancer were more likely to report experiencing depression or anxiety, and the odds of reporting anxiety and depression were 2.18 and 3.66 greater for those with upper gastrointestinal cancer compared to those with no cancer history. Upper gastrointestinal cancer survivors can experience physical symptoms, changes in eating behaviors, and physical functioning leading to appreciable impacts on quality of life [22, 23], which in turn may increase risk of depression and anxiety [23, 24]. However, due to small sample sizes in this diagnosis group, this result should be interpreted with caution and warrants further research.

Participants with a history of cancer were more likely to report difficulties with concentrating than participants without a history of cancer, and those with colorectal or breast cancer diagnosis were at particular risk, as were women. These results are largely consistent with previous studies employing large nationally representative samples [15, 25], and a recent review of longitudinal studies of self-reported cognitive impairment after chemotherapy which indicated between 11 and 37% of survivors experienced significant cognitive decline after initiating chemotherapy [17].

For all mental health outcomes, we did not find an effect of time since diagnosis, with a relatively consistent proportion of participants reporting difficulties with anxiety, depression, and concentration after treatment. Mental health concerns may remain relatively stable in the early years after diagnosis. For example, in the first 12 months after diagnosis, prevalence of anxiety and depression in a population-based sample was relatively stable at around 20% and 13% respectively [26]. Longitudinal studies with a longer follow-up suggest some improvement in symptoms of depression [14, 27, 28], and self-reported cognitive difficulties [17], as time post treatment increases. However, there appears to be a proportion of survivors that continue to experience significant mental health symptoms that impact on their quality of life [29], and ability to return to usual roles (e.g., work) [30].

Consistent with previous research, we found strong correlations between mental health outcomes. Anxiety and depression were highly correlated while difficulty with concentration was moderately correlated with both anxiety and depression. These results add to the growing body of literature demonstrating interrelatedness of anxiety, depression, and cognitive symptoms [9, 12, 16, 31]. However, our study and most previous studies have been cross-sectional, making causal inferences impossible about whether one symptom precedes another or whether they are co-occurring. Furthermore, it is possible that fatigue, sleep quality, and other psychological factors such as stress and loneliness contribute to reporting mental health symptoms [8, 32–34]. Given many of these symptoms are amenable to intervention [35], further research is needed to understand the interrelatedness, any potential confounds, and causal directions of psychological symptoms experienced during survivorship to offer tailored support with the potential to improve quality of life overall.

There are several limitations worth noting. This study relied on single-item measures of anxiety, depression, and cognitive symptoms. While a single question covering mental health symptoms is brief and potentially mimics clinical encounters, they may miss important impacts. For example, only difficulties regarding concentration were assessed, yet previous research has consistently demonstrated survivors often report difficulties with memory and other cognitive abilities [36]. Further research investigating the utility of single-item questions for screening for mental health concerns is warranted [37]. Our sample had a median age at diagnosis of 51 years, over-representation of non-Hispanic White, and well-educated participants, as well as small sample sizes in some diagnostic groups. As such, our results may not generalize to all cancer survivors and should be interpreted cautiously for survivors of respiratory, upper gastrointestinal, and hematological cancers. Furthermore, we had no data regarding stage of disease and treatment which may also impact symptom severity.

We report on data from a large national sample of participants using single-item measures of three commonly experienced mental health concerns. We found no evidence of difference in the prevalence of anxiety and depression between participants with and without a history of cancer, but those with a history of cancer were more likely to report difficulties with concentration. Incidence of these symptoms was stable in the years after cancer diagnosis. Anxiety, depression, and difficulties with concentration were strongly related; however, further research is needed to explore the causal direction of these correlations, so that interventions may be appropriately targeted.

## Supplementary Information

Below is the link to the electronic supplementary material.

Supplementary file1 (DOCX 25 KB)

## Data Availability

All data are available for download from the NHANES website. Centers for Disease Control and Prevention (CDC). National Center for Health Statistics (NCHS). National Health and Nutrition Examination Survey Data. Hyattsville, MD: U.S. Department of Health and Human Services, Centers for Disease Control and Prevention, https://wwwn.cdc.gov/nchs/nhanes/Default.aspx.
