# Supplementary material for: Anxiety, depression, and concentration in cancer survivors: National Health and Nutrition Examination Survey results
Source: Support Care Cancer. 2023 Apr 15;31(5):272. doi: 10.1007/s00520-023-07710-w (PMC10105664; doi:10.1007/s00520-023-07710-w)
Supplement: Supplementary file 1 — Supplementary file1 (DOCX 25 KB) [file 520_2023_7710_MOESM1_ESM.docx]

**Supplementary Appendix**

**Table S1.** Propensity score sensitivity analysis of association between history of cancer and experiencing negative mental health outcomes in participants of NHANES, 2015-2018, (N = 10,337). Results of logistic regression analysis are presented with adjusted odds ratios (OR).

| **Outcome** | **Adjusted^a^ OR: Primary Analysis** | **Adjusted^b^ OR: Sensitivity Analysis** |
| --- | --- | --- |
| **Anxiety^c^** | 1.12 (0.87, 1.43) | 1.13 (0.88, 1.46) |
| **Depression^d^** | 1.10 (0.86, 1.41) | 1.10 (0.86, 1.41) |
| **Concentration difficulties** | 1.38 (1.00, 1.90)* | 1.37 (0.99, 1.89) |

* p < 0.05

^a^Adjusted for gender, age, race/ethnicity, and education, reference group is no cancer history

^b^Adjusted by propensity score stratification, reference group is no cancer history

^c^Self-reported anxiety occurring daily, weekly, or monthly

^d^Self-reported depression occurring daily, weekly, or monthly

**Table S2. Percentage of participants reporting each mental health outcome, and co-occurrence of mental health outcomes**

| **Outcome** | **Cancer History (N = 691)** | **No Cancer History**  **(N = 9,646)** |
| --- | --- | --- |
| **Anxiety, N (%)^b^** |  |  |
| Daily | 121 (17.8%) | 1,427 (15.8%) |
| Weekly | 97 (14.4%) | 1,355 (17.9%) |
| Monthly | 90 (13.6%) | 1,182 (13.2%) |
| A few times a year | 228 (34.8%) | 3,353 (33.4%) |
| Never | 155 (19.4%) | 2,329 (19.6%) |
| **Depression, N (%)^b^** |  |  |
| Daily | 62 (7.3%) | 498 (4.6%) |
| Weekly | 44 (6.0%) | 655 (6.6%) |
| Monthly | 71 (6.7%) | 756 (8.5%) |
| A few times a year | 206 (34.6%) | 3,122 (33.9%) |
| Never | 308 (45.3%) | 4,615 (46.3%) |
| **Concentration difficulties, N (%)^b^** | 94 (11.3%) | 934 (9.0%) |
| **At Least Two Outcomes^a^, N (%)^b^** | 177 (19.6%) | 1,906 (20.1%) |
| **All Three Outcomes^c^, N (%)^b^** | 558 (5.5%) | 59 (7.9%) |
| **Concentration difficulties and Depression, N (%)^b^** | 63 (8.4%) | 595 (5.8%) |
| **Concentration difficulties and Anxiety, N (%)^b^** | 74 (9.3%) | 731 (7.3%) |
| **Depression and Anxiety, N (%)^b^** | 158 (17.7%) | 1,696 (18.0%) |

^a^Participants reporting at least two out of three mental health outcomes

^b^Percentages are survey-weighted

^c^Participants reporting anxiety, depression and concentration difficulties.
